# Navigating the Complex Solid Form Landscape of the Quercetin Flavonoid Molecule

**DOI:** 10.1021/acs.cgd.3c00584

**Published:** 2023-07-13

**Authors:** Panayiotis Klitou, Emmanuele Parisi, Simone Bordignon, Federica Bravetti, Ian Rosbottom, Marzia Dell’Aera, Corrado Cuocci, Michele R. Chierotti, Angela Altomare, Elena Simone

**Affiliations:** † School of Food Science and Nutrition, Food Colloids and Bioprocessing Group, University of Leeds, Leeds LS2 9JT, UK; ‡ Department of Applied Science and Technology (DISAT), Politecnico di Torino, Torino I-10129, Italy; § Dipartimento di Chimica I.F.M, Università degli Studi di Torino, Via P. Giuria 7, Torino I-10125, Italy; ∥ School of Chemical and Process Engineering, University of Leeds, Woodhouse Lane Leeds LS2 9JT, UK; ⊥ Institute of Crystallography IC − CNR, via Amendola 122/O, Bari I-70126, Italy

## Abstract

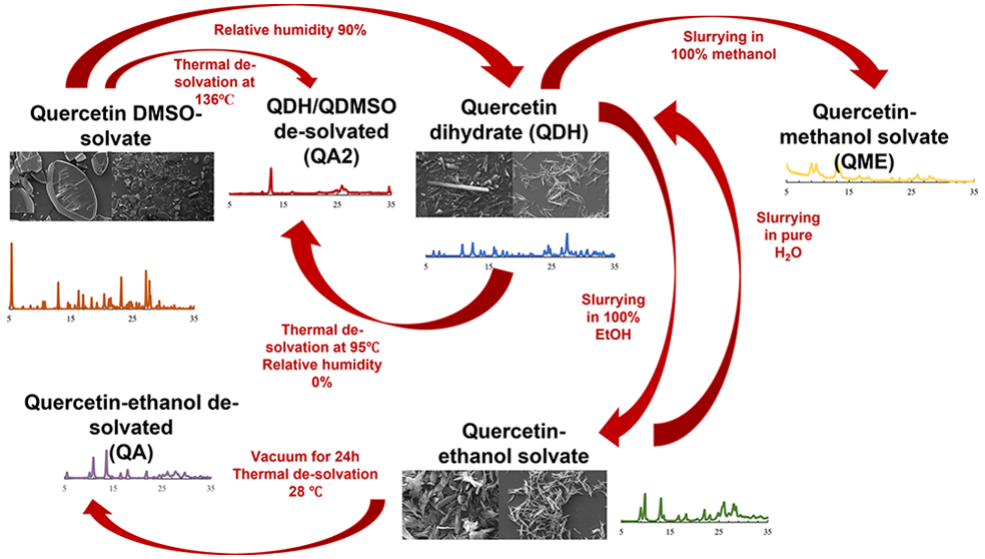

Quercetin, a naturally occurring bioflavonoid substance widely used in the nutraceutical and food industries, exists in various solid forms that can have different physicochemical properties, thus impacting this compound’s performance in various applications. In this work, we will clarify the complex solid-form landscape of this molecule. Two elusive isostructural solvates of quercetin were obtained from ethanol and methanol. The obtained crystals were characterized experimentally, but the crystallographic structure could not be solved due to their high instability. Nevertheless, the desolvated structure resulting from a high-temperature treatment (or prolonged storage at ambient conditions) of both these two labile crystals was characterized and solved via powder X-ray diffraction and solid-state nuclear magnetic resonance (SSNMR). This anhydrous crystal structure was compared with another anhydrous quercetin form obtained in our previous work, indicating that, at least, two different anhydrous polymorphs of quercetin exist. Navigating the solid-form landscape of quercetin is essential to ensure accurate control of the functional properties of food, nutraceutical, or pharmaceutical products containing crystal forms of this substance.

## Abstract 2

Study of physicochemical and crystallographic properties of different quercetin solid forms.

## Introduction

**Scheme 1:**
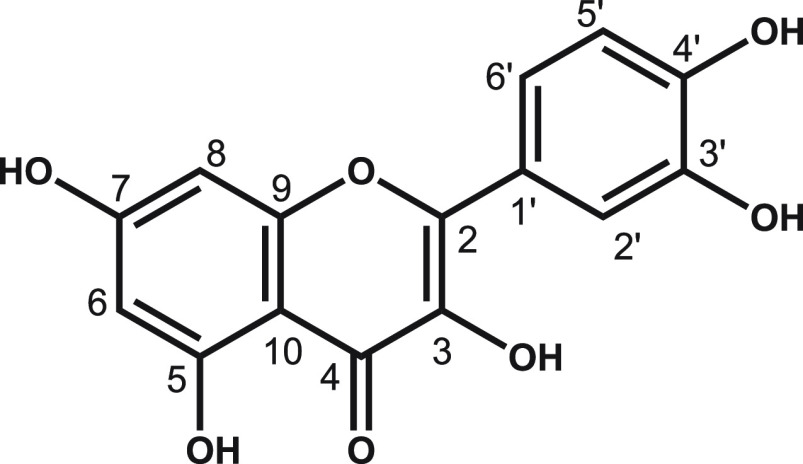
Molecular Structure of Quercetin, with C Atom Numbering

Quercetin, 2-(3,4-dihydroxyphenyl)-3,5,7-trihydroxy-4*H*-chromen-4-one (Scheme 1), is a major dietary flavonol found in many fruits and vegetables, including onions, tomatoes, apples, and berries.^1,2^ It belongs to a group of plant metabolites, named flavonoids, which are thought to provide health benefits through cell signaling pathways and antioxidant effects.^3^

The quercetin molecule consists of a pyrone ring and phenyl ring, which constitute the hydrophobic part of the molecule and can form hydrophobic interactions such as van der Waals forces of attraction.^2,4^ The hydrophilic part of the molecule consists of five hydroxyl groups that determine the molecule’s biological activity and can act as hydrogen-bond acceptors and/or donors, as well as an ether and carbonyl group acting as acceptors for both intramolecular and intermolecular hydrogen bonding.^4−7^

Quercetin has stimulated considerable interest in recent years, and it is the most extensively studied flavonoid, due to its significant association between dietary consumption and various health benefits, including antioxidant, anti-inflammatory, and antitumoral activities.^1,2,4,8,9^ Due to this wide range of health benefits and biological effects, quercetin finds a multitude of applications in the food and nutraceutical industries.^2^ Quercetin dihydrate is marketed as a dietary supplement in a capsule form, to help improve anti-inflammatory and immune response.^10^

As several solvents and processing conditions can be used in the manufacturing of crystalline quercetin as well as for its application, it is important to have a clear understanding of the solid-form landscape of the compound.^11−13^ A thorough knowledge of the crystal forms of quercetin and the transformation conditions between them is essential to design storage conditions, avoid any unexpected transformations during manufacturing, and ensure accurate control of the functional properties of products containing quercetin.

**Figure 1:**
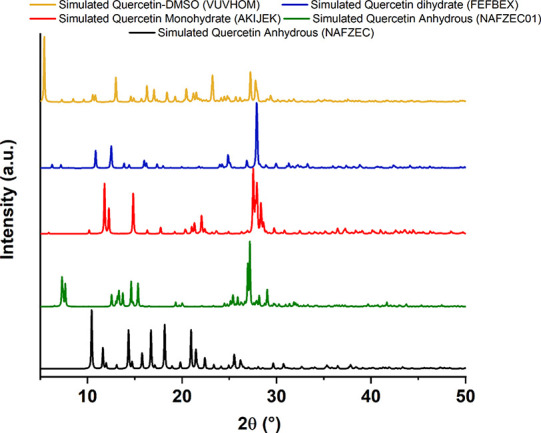
Simulated PXRD patterns for some solved quercetin structures (CCDC identifier shown in brackets).

A Cambridge Crystallographic Data Centre (CCDC) search on quercetin crystal forms yields a vast range of structures. These include solvates and hydrates, cocrystals, and cocrystal solvates, such as a quercetin-DMSO solvate, quercetin-isonicotinamide, quercetin-praziquantel, and quercetin-theophylline cocrystals, just to name a few.^14−17^Figure 1 summarizes briefly the most common solid forms of quercetin reported in literature, showing the powder X-ray diffraction (PXRD) patterns for the two deposited anhydrous quercetin forms, the two hydrates, and the DMSO solvate (QDMSO) recently solved by our group.

The more stable and commercially available solid form of quercetin is the dihydrate (space group *P*1̅, triclinic). This was solved in 1985 by Rossi et al. (CCDC identifier: FEFBEX) from a single crystal obtained by evaporation of an aqueous ethanol solution and also reported by Jin et al. in 1989 (CCDC identifier: FEFBEX01) by evaporation of an aqueous 1-propanol solution.^1,18^ Quercetin monohydrate (space group *P*2_1_/*c*) was first reported in 2011 by Domagata et al. who determined its PXRD pattern and applied the multipolar atom model to analyze the structure in terms of its geometry, molecular packing, and intra- and intermolecular interactions (CCDC identifier: AKIJEK).^19^ The monohydrate structure was nucleated from an acetonitrile solution; however, the exact experimental procedure remain unclear.

Information on the anhydrous polymorphs of quercetin is confusing, due to the difficulty in obtaining crystals of sufficient size and quality for single-crystal X-ray diffraction. Such issue is well highlighted in literature, and it is the reason why the structure of anhydrous quercetin and its more or less stable polymorphs still remains poorly understood.^1,18−20^ In 2004, Olejniczak et al. confirmed the existence of an anhydrous form by several experimental techniques (PXRD, DSC, TGA, and SSNMR) and DFT calculations.^20^ However, the authors did not deposit any structure in the CCDC. Filip et al. in 2013 followed a multitechnique approach, combining PXRD data with information from SSNMR and molecular modeling to elucidate the conformation of quercetin in the anhydrous structure and gain insight into the relationship between the hydrogen-bond network and crystal packing pattern.^21^ In 2016, the first structure of anhydrous quercetin (CCDC identifier: NAFZEC) was deposited by Vasisht et al., who solved it using computational analysis of PXRD data, and indicating that quercetin crystallizes in an orthorhombic anhydrous form with four molecules per unit cell, and space group *Pna*2_1_.^6^ However, the data and lattice parameters for the anhydrous quercetin suggested by Vasisht et al. are reported several times in literature to be problematic and present large deviations from further lattice and geometry optimizations of the structure.^21,22^ In 2016, Miclaus et al. reported the presence of two weak and highly unstable methanol and ethanol solvates, which result from quercetin recrystallization from the respective solvents and occur in mixtures with the anhydrous form.^23^ It is reported that, in the two solvates, the solvent molecules are weakly hydrogen-bonded to the quercetin molecules and serve as intermediates in the transformation to an anhydrous quercetin form. The transformation was studied using SSNMR, and the PXRD patterns of the weak solvates and their resulting anhydrous forms were obtained.^23^ However, the anhydrous quercetin PXRD pattern does not match with that of anhydrous quercetin previously solved by Vasisht et al. Finally, in 2020, another anhydrous quercetin structure was determined (CCDC identifier: NAFZEC01) from PXRD data by Maciołek et al.^24^ This anhydrous quercetin form was an intermediate product of the thermal degradation of sodium 3,3′,4′,5,7-pentahydroxyflavon-5′-sulfonate tetrahydrate at 285 °C and was recrystallized from its molten phase. The structure was solved in the *C*2/*c* space group, with four symmetrically independent quercetin molecules in the unit cell. The PXRD pattern of the *C*2/*c* anhydrous form is different from the previously reported structure by Vasisht et al.

In this work, we explored and clarified the structure of anhydrous quercetin and its polymorphs. Furthermore, we studied the relative stability of such crystal forms and the possible crystallization pathways. As part of this investigation, we have found and experimentally characterized two elusive solvates of methanol and ethanol, which were obtained as intermediates during the crystallization of anhydrous quercetin. In summary, a wide range of solid-state characterization techniques were used to determine the solid-form landscape of quercetin, understanding relative stability and kinetics of polymorphic transformation in the solid state. A good knowledge of this information can guide the choice of crystallization parameters to target a particular form of quercetin and ultimately lead to faster product and process development.

## Experimental Section

### Materials

Quercetin dihydrate (QDH) with a purity of 97% was obtained from Alfa Aesar (Port of Heysham Industrial Park, Lancashire, England); ethanol, 99.98%, was purchased from VWR chemicals; and methanol, 99.98%, was purchased from Sigma-Aldrich. 2-Propanol, 99.5%, was obtained from Sigma-Aldrich. Water purified by treatment with a Milli-Q apparatus was used for all the experiments.

### Slurrying of QDH in Ethanol-Water Solvent Mixtures (QE)

Slurries of quercetin in ethanol-water solvent mixtures were prepared by adding 4.0 g of QDH in 100 g of 100, 90, 85, 75, 60, and 15% (w/w) ethanol-water solvent mixtures. The temperature of the slurry was kept constant at 20 °C using a Tamson TLC2 recirculating chiller. The slurry was stirred using magnetic stirring at approximately 300 rpm for 48 h. The solid samples removed from the slurry were filtered using a Büchner flask, funnel, and filter paper to remove the solvent. The samples were allowed approximately 24 h to dry completely.

Approximately 10 mL of supernatant solution in the slurry experiments carried out in 100% ethanol was transferred to several Petri dishes together with seed crystals from the same slurry. This was done to promote growth of the seeds by evaporation and study more in detail the morphology of the obtained crystals, which we named QE. The Petri dishes were covered with Parafilm with holes to allow slow, controlled evaporation of the ethanol.

### Slurrying of QDH in Methanol (QME)

QDH was slurried in 100% methanol following the same methodology as for 100% ethanol. The solid samples were filtered and allowed approximately 24 h to dry.

### Scanning Electron Microscopy (SEM)

The crystal morphology of the QE crystals was determined using SEM. The dry samples were imaged using a Carl Zeiss EVO MA15 scanning electron microscope. Samples were arranged on Leit tabs attached to SEM specimen stubs, and an iridium coating was applied before measurement. Samples from the 100% ethanol slurry and from the growth experiments on the Petri dishes were imaged.

### Thermogravimetric Analysis Coupled with Differential Scanning Calorimetry (DSC/TGA)

TGA and DSC experiments were performed on a Mettler Toledo TGA/DSC 3+ Stare System equipment. The samples (around 10–15 mg) were placed in 70 μL aluminum pans, covered with a lid, and heated from 20 to 500 °C at a heating rate of 10 °C min^–1^. Nitrogen was used as purge gas at 50 mL min^–1^. Measurements were repeated three times. The samples were filtered the day before the analysis and left to dry overnight.

### X-Ray Diffraction (SAXS/WAXS, PXRD, VT-PXRD)

The small and wide-angle X-ray scattering (SAXS/WAXS) data were collected on a SAXSpace instrument (Anton Paar GmbH, Graz, Austria) equipped with a Cu anode that operates at 40 kV and 50 mA (λ = 0.154 nm). The PXRD data were collected on: (1) a Panalytical X’Pert PRO, which was set up in Bragg-Brentano mode, using Cu Kα radiation (λ = 1.54184 Å), in a scan between 5° and 50° 2θ with a step size of 0.032° (2θ) and time per step of 25 s; (2) a Rigaku Rint2500 rotating Cu anode source, working at 50 kV and 200 mA in Debye–Scherrer geometry. The latter diffractometer is equipped with an asymmetric Johansson Ge (111) crystal to select the monochromatic Cu Kα1 radiation (λ = 1.54056 Å) and the silicon strip Rigaku D/teX Ultra detector. Data were collected from 5° to 80° (2θ) with a 0.02° (2θ) step size and counting time of 6 s/step. The powder was introduced in a glass capillary of 0.5 mm in diameter and mounted on the axis of the goniometer. The capillary was rotated during the measurement to improve the randomization of the orientation of the individual crystallites to reduce the effect of possible preferred orientation.

The Variable Temperature PXRD (VT-PXRD) data were collected on the Panalytical X’Pert PRO, and the temperature was increased from 20 to 90 °C at a rate of 10 °C min^–1^. The analysis of the crystal packing was performed using the program Mercury, version 2022.3.0.^25^

### QE Mass Loss over Time Experiments

#### Dynamic Vapor Sorption (DVS) Experiment

A 50 mg sample of QE in 100% ethanol slurry was placed on a DVS pan, and the mass change over a period of 20 h was monitored, at a constant temperature of 20 °C and a relative humidity (RH) of 20%. The DVS experiments were performed on a Surface Measurement Systems DVS Resolution equipment.

#### Monitoring Sample Mass of QE over Time

A sample of QE was filtered, and the solid was placed on a plastic Petri dish and left uncovered at ambient conditions. The mass of the sample was measured once a day for 6 days with a lab balance to observe any mass changes.

### Stability Studies for the QE Crystals

The thermodynamic stability of QE was determined by measuring the SAXS/WAXS patterns of QE samples treated under different conditions. The samples tested include: 4-week-old and 16-month-old samples of QE left at room-temperature conditions in the laboratory, a sample of QE slurried in pure water for 24 h and magnetically stirred at 300 rpm, and a sample of QE that was treated in a vacuum oven at 0 mbar for 24 h.

### Solid-State NMR Spectroscopy

Solid-state ^13^C CPMAS NMR spectra were acquired with a Bruker Avance II 400 Ultra Shield instrument, operating at 400.23 and 100.63 MHz, for ^1^H and ^13^C nuclei, respectively. The powder sample was packed into cylindrical zirconia rotors with a 4 mm o.d. and 80 μL volume. A certain amount of sample was collected from each batch and used without further preparations to fill the rotor. The ^13^C CPMAS spectra were acquired at a spinning speed of 12 kHz, using a ramp cross-polarization pulse sequence with a 90° ^1^H pulse of 3.60 μs, a contact time of 3 ms, a recycle delay ranging from 1 to 10 s, and a number of scans between 100 and 820, depending on the sample. A two-pulse phase modulation (TPPM) decoupling scheme was used, with a radiofrequency field of 69.4 kHz. The ^13^C chemical shift scale was calibrated through the methylenic signal of external standard α-glycine (at 43.7 ppm).

### Calculations

Crystal structures were optimized using the Quantum Espresso suite (v. 6.4.1),^26^ employing the projector-augmented wave (PAW) approach, with the nonlocal vdW-df2 method^27^ and B86r functional^28^ with the SSSP set of pseudopotentials.^29^ An energy cut-off of 60 Ry was used. The experimental and optimized crystal structures were visualized and compared using the CSD program Mercury. The RMSD20 was calculated with the “crystal packing similarity” utility, considering a cluster of 20 molecules and a tolerance value of 20% on angles and distances.

### Crystal Structure Solution via Powder X-Ray Diffraction (PXRD)

The *ab initio* solution and structure refinement process were automatically performed by the EXPO software,^30^ a package capable of carrying out the following steps: (a) determination of unit cell parameters and identification of space group, (b) structure solution by direct methods and/or real-space approach, and (c) structure model refinement by the Rietveld method.^31^ The first low-angle well-defined peaks in the experimental diffraction pattern were selected using a graphical peak selection tool and actively used for indexing via N-TREOR09^32^ and DICVOL04^33^ programs embedded in EXPO. The space group determination was determined on the evaluation of the systematic absences.

Each structure was solved with a real-space method based on the simulated annealing algorithm implemented in EXPO. The starting model was derived from the crystal structure of the anhydrous quercetin polymorph, refcode NAFZEC,^6^ obtained from CSD,^34^ and the geometry optimization was achieved by the program MOPAC.^35^ The simulated annealing algorithm was run 20 times under Linux workstation in default mode and in parallel calculations over 20 CPUs. The best solution with the lowest cost function value was selected. The criterion to accept the solution was also based on the soundness of crystal packing. The solution obtained by the direct-space method was also confirmed by direct methods.

Density-functional theory (DFT) geometry optimization with Quantum ESPRESSO^36^ was only performed on hydrogen atoms to improve their positions. The derived structure was refined by the Rietveld method. Restraints were applied to bond distances to stabilize the refinement. All H atoms bonded to C atoms were treated as riding under the constraint on atomic displacement parameters *U*_iso_(H) = 1.2 · *U*_iso_(C). Peak shape was modeled using the Pearson VII function. The atomic displacement parameters were refined isotropically and constrained to have the same value for atoms of the same chemical species.

### Solubility Measurements of Quercetin Anhydrous (QA), Quercetin-Methanol (QME), Quercetin-Ethanol (QE), Quercetin Dihydrate (QDH), and Quercetin Anhydrous from Desolvation of QDMSO (QA2)

The Crystal16 equipment (Technobis) was used to determine the solubility of quercetin solid forms. Isopropanol was used as a reference solvent. In the Crystal16, clear points of eight 1 mL stirred vials can be measured in parallel and automatically, based on the value of the turbidity. The bottom stirring speed was set at 800 rpm. The temperature at which the suspensions become clear solutions by heating (rate of 0.3 °C min^–1^) was taken as the saturation temperature of the measured samples.

## Results and Discussion

### Slurrying of QDH in Ethanol-Water Solvent Mixtures and Methanol

**Figure 2:**
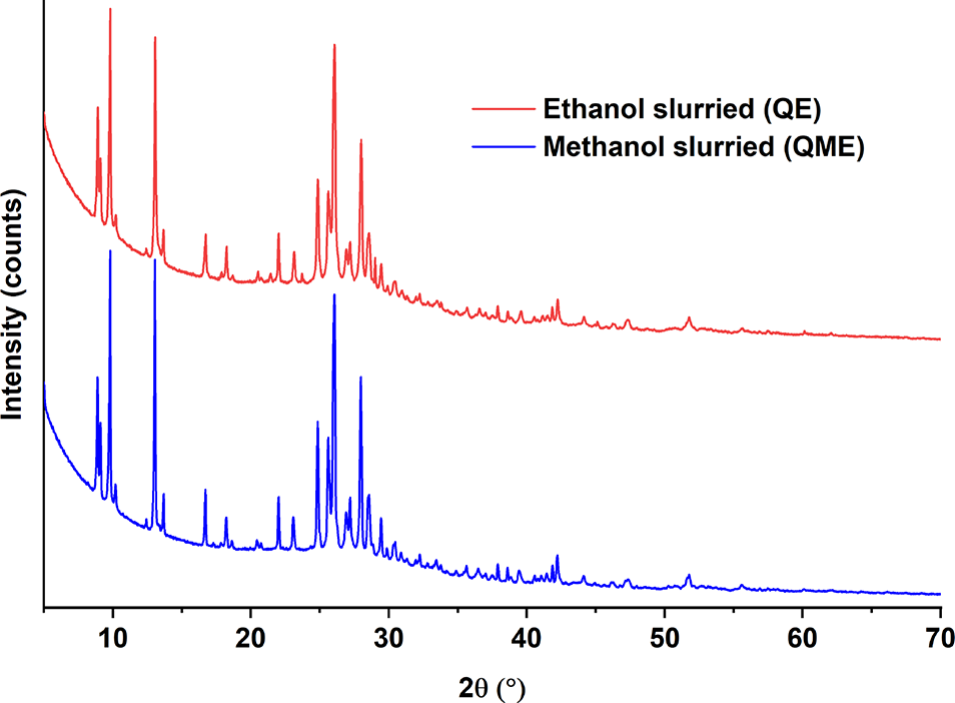
PXRD patterns for quercetin slurried in ethanol (QE) and in methanol (QME).

The solid crystals from the various ethanol-water solvent mixtures after slurrying were tested using SAXS/WAXS to identify the solid form (Supporting Information, Figure S1). The SAXS/WAXS patterns for the solid samples from 15 to 90% (w/w) ethanol slurries were identical to that of quercetin dihydrate. This means that the stable solid form of quercetin for those ethanol-water solvent mixtures is the dihydrate. QDH as purchased was also tested using SAXS/WAXS and is shown in Figure S1 for comparison. The solid taken from the 100% ethanol slurry exhibits a different PXRD pattern, which does not correspond to either QDH or its desolvated form or any other deposited quercetin structure.^1,6,14,19,24^ However, the pattern looks identical to the pattern previously reported by Miclaus et al., who described it as a weak quercetin-ethanol solvate.^23^ This could be a case of a solvent-mediated polymorphic transformation, in which QDH dissolves in pure ethanol and recrystallizes as a weak solvate crystal structure that seems to form interactions with ethanol itself. This ethanol-slurried sample will be referred to as QE in the manuscript. The quercetin sample obtained from slurrying QDH in pure methanol (QME) was also studied and its PXRD pattern collected. In Figure 2, it can be observed that the QE and QME patterns are almost identical. For better resolution, PXRD data of QE and QME were collected on the Rigaku Rint2500 instrument. The QE pattern exhibits main peaks at 2θ angles of 4.54°, 8.92°, 9.80°, 13.08°, 24.84°, 26.06°, and 28.00°.

The fact that QE and QME display the same diffraction peaks is particularly interesting, as it could indicate that these two quercetin forms are isostructural. Isostructural crystal structures have been previously shown in the literature to share very similar XRD patterns resulting from similar crystal structures and packing patterns, but different cell dimensions and chemical composition.^37^ This type of behavior would not be a surprise as the methanol and ethanol molecules are very similar, each containing a hydroxyl group of very similar electronegativity, and ethanol only being slightly bigger in size just by a methyl group. It is, therefore, expected that the type and strength of intermolecular interactions that they would form with the quercetin molecules would not differ greatly, and this should result in similar packing arrangements in the lattice. It is worth noticing that slurrying QDH in isopropanol did not result in the formation of a hypothesized solvate structure, perhaps due to the larger size of this molecule.

It is interesting to notice that QE is only obtained from slurrying QDH in pure ethanol, and above 10% (w/w) of water in the solvent resulted in QDH being the most stable form. It seems that the interaction with the ethanol molecules in solution is weaker than with the water molecules, and this could possibly be due to the bulkier size of the ethanol molecule compared to water, which is impacting the strength of the hydrogen-bond interactions with the quercetin molecules and, thus, making it unable to offer the same degree of stabilization of the lattice as water molecules.^14^ In our previous publication, it was shown how the water molecules in the QDH lattice satisfy Kitajgorodskij’s rule for the hydrogen-bond interactions, leading to a close-packed structure of higher relative stability compared to the previously known monohydrate and anhydrous quercetin forms.^38^ Therefore, it is not a surprise that, even at a low ratio of water in the solvent mixture, QDH is the stable form. This phenomenon has been observed and reported in literature before.^39,40^ When the water activity of the solvent mixture exceeds a critical water activity value, the hydrate form of the crystal is the thermodynamically stable form. However, when the water activity of the solvent mixture is below the critical value (in our system, the critical water activity corresponds to a water concentration less than 10%(w/w)), then the solute molecules interact primarily with the other solvent molecules (i.e., ethanol in our system), excluding the water molecules from the lattice.^39,40^ Similar studies have been conducted in the past for carbamazepine and theophylline, to understand the relation between solvent water activity and the hydration state of the solid phase that crystallizes, and generating three-component phase diagrams for those systems.^41,42^ It is also reported that the critical water activity depends on factors such as the temperature, pressure, and the nature of the solute and solvent.

Overall, the slurrying experiments demonstrate that, for applications where QDH is desired, the use of mixtures of ethanol and water to increase the solubility of quercetin in solution is safe, as long as the ethanol ratio in solution is 90% (w/w) or lower. Pure ethanol will result in the formation of a different quercetin structure.

### Scanning Electron Microscopy (SEM)

**Figure 3:**
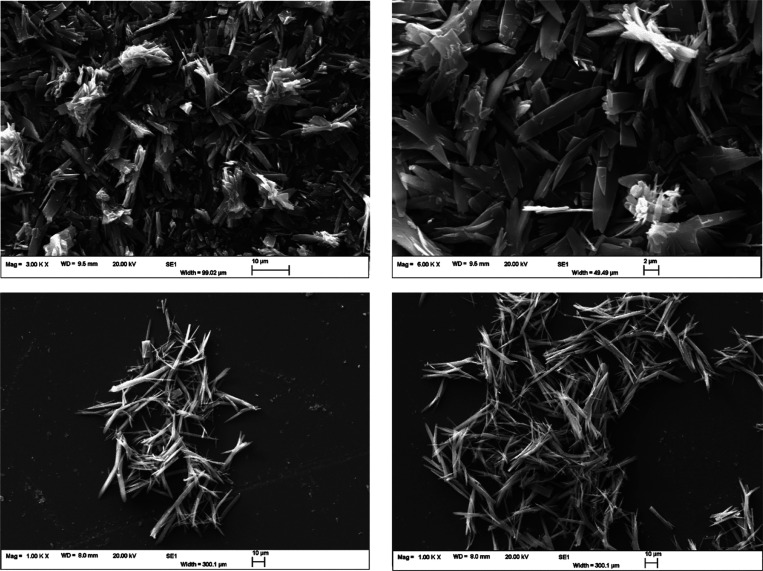
SEM images of the QE crystals from the 100% ethanol slurry (top) at 3–6 K X magnifications and from the growth experiments on Petri dishes (bottom) at 1 K X magnifications.

Images of QE crystals are shown in Figure 3.

The SEM images show a needle morphology for the QE crystals, although the crystals from the 100% ethanol slurry are flakier and smaller in size compared to those grown on the Petri dishes. These latter ones appear bigger in size, between 20 and 40 μm, and have a higher aspect ratio compared to the ones obtained directly from the slurry. It should be noted that this morphology is very similar to that of the QDH crystals, which also exhibit a needle-like shape. For comparison, SEM images of the morphology of the QDH crystals are shown in the Supporting Information, Figure S2.

### Thermal Stability of QE and QME

#### Thermogravimetric Analysis Coupled with Differential Scanning Calorimetry (TGA/DSC)

**Figure 4:**
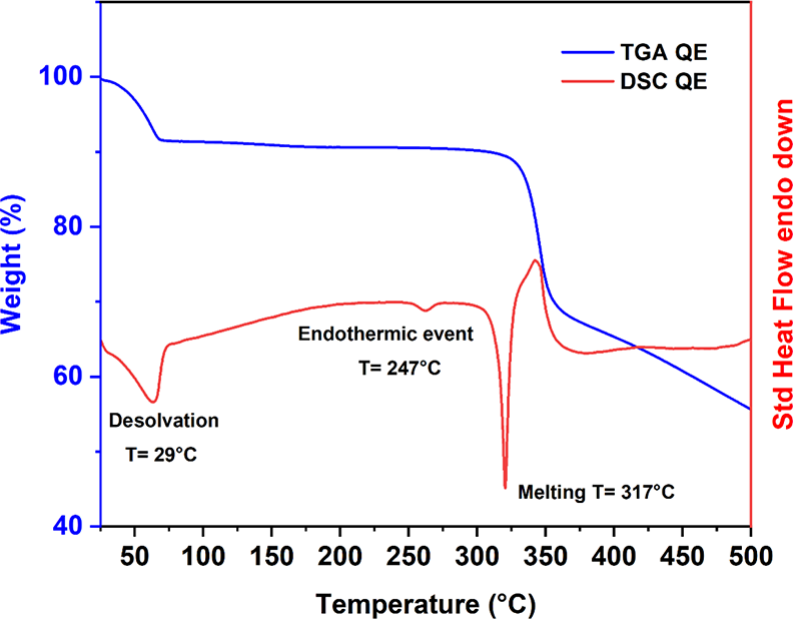
DSC curve of QE in red. TGA curve of QE in blue.

The thermal stability of the QE structure was studied to assess under what conditions of temperature the sample undergoes changes in mass or heat flow. The results for the TGA coupled with DSC are shown in Figure 4.

Observing the TGA curve, there is a loss in mass of about 6.2%, starting at an onset temperature of 28.5 °C and finishing at approximately 70 °C. This loss in mass is accompanied by an endotherm as seen on the DSC curve. The loss could be attributed either to free ethanol evaporating from the wet solid (e.g., if the sample was not completely dry after being left to dry overnight), or to a desolvation process, where the ethanol molecules leave the crystal lattice.

To confirm which of the two was the reason for the weight loss, the mass of ethanol was monitored in two different experiments: a DVS experiment, where the mass was monitored for 24 h under controlled relative humidity and temperature conditions, and a mass-loss over time experiment, where the mass of a QE sample left at ambient conditions (approximately 20 °C) was monitored for several days. The data for these experiments are shown in the Supporting Information, Figures S3 and S4. Both experiments confirmed that the mass of QE does not change considerably after the first day of drying. More specifically, the mass of a sample of QE after one day of drying to the sixth day just decreased by 0.8%. This confirms that, during the TGA/DSC experiment, it is very unlikely that the sample lost 6.2% of its mass due to an incomplete drying process. Hence, the thermal event observed in the DSC should be associated to a desolvation event.

The theoretical mass loss for a stoichiometry of one molecule of ethanol to one molecule of quercetin is calculated to be 13.2%. The observed loss was much less than that, almost half, and there was also significant variability in the mass loss between the different measurements of crystals from the same batch. This further suggests that the measured QE sample was highly unstable, and that what was actually measured with the TGA/DSC is a mixture of QE and an anhydrous form of quercetin. Miclaus et al. also emphasized in their paper the difficulty in obtaining a pure form of QE due to its low stability.^23^ There is no further loss in mass after the endset temperature of 70 °C and before the quercetin chemical decomposition at 335.3 °C.

The melting point of QE occurs at a sharp temperature of 317 °C, which agrees with the melting point of quercetin, starting either from QDH nor QDMSO forms.^14^ However, it is interesting to note that a small endotherm occurs just before melting, at an onset temperature of 247.3 °C. This endotherm is obtained neither for QDH or QDMSO, and it is probably due to a structural rearrangement that occurs in the crystal lattice before melting. If the ethanol molecules are weakly hydrogen-bonded to the quercetin molecules, they might have escaped the lattice during the thermal desolvation event with a conformational rearrangement of the whole structure. Therefore, it is possible that, during that small endothermic event, the quercetin molecules rearrange to attain a more stable conformation with a melting point equivalent to that of the structure obtained from heating both QDH and QDMSO. The quantitative data from the TGA/DSC measurements are summarized in Table 1.

**Table 1:** TGA/DSC Thermal Analysis Data for QE

assumed stoichiometry	1:1
theoretical weight loss (%)	13.2%
observed TGA weight loss (%)	6.2 ± 2.4
guest loss temp. (°C)	28.5 ± 5.1
Δ*H* for guest loss (Jg^–1^)	–102.3 ± 35.5
structural rearrangement temp. (°C)	247.3 ± 7.2
Δ*H* for structural rearrangement (Jg^–1^)	–8.2 ± 1.0
melting temp. (°C)	316.5 ± 0.8
Δ*H* for melting (Jg^–1^)	–125.7 ± 16.0
decomposition temp. (°C)	335.3 ± 7.3

**Figure 5:**
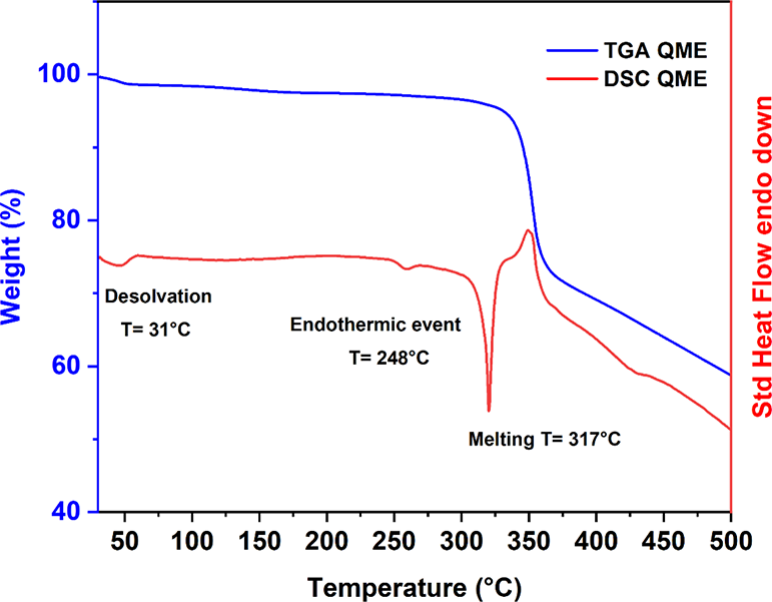
DSC and TGA curves for QME.

The thermal behavior of QME was also studied by TGA/DSC, and the results are illustrated in Figure 5. The thermal events observed for QME are very similar to those of QE. The structure exhibits an endothermic event at an onset temperature of 31 °C, accompanied by a loss of 2.3% of its mass, which could be linked to a desolvation step. Furthermore, a similar small endotherm, possibly due to a structural rearrangement just before melting, is observed at approximately 248 °C, similarly to QE. Finally, the structure melts at a temperature of 317 °C, agreeing with the melting temperature of quercetin. For both QE and QME, the thermal analysis seems to highlight the formation of a weak solvate with the loss of a nonstoichiometric amount of ethanol and methanol molecules.

#### Variable Temperature Powder X-Ray Diffraction (VT-PXRD)

**Figure 6:**
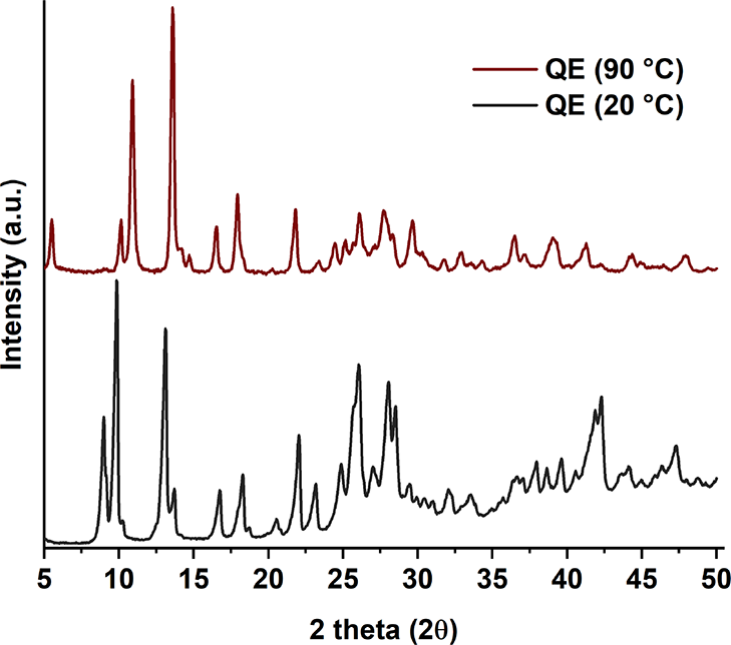
VT-PXRD patterns for QE at 20 and 90 °C.

To verify the presence of structural changes in QE between 28 and 70 °C, the PXRD pattern of this sample was measured at 90 °C. The results are shown in Figure 6. From the PXRD data, it is evident that there is a change in the structure, as the main peaks are different before and after heating. The two main peaks of QE (20 °C) at 8.9° and 9.8° disappear, and two new peaks appear for QE (90 °C) at 10.2° and 10.9°. Furthermore, the main peak of QE (20 °C) at 13.0° disappears and another one at 13.6° appears for QE (90 °C).

Combining the data from the TGA curve and QE (90 °C) pattern, it can be confirmed that this pattern belongs to an anhydrous form of quercetin, as no further loss in mass appears to be occurring at any higher temperature before decomposition. From now on, we will refer to this anhydrous form as QA.

Moreover, these data suggest that the initial sample of QE at 20 °C could already contain a small amount of desolvated form, as the QE (20 °C) pattern contains small peaks at 2θ angles of 10.2° and 13.6°, which increase in intensity in the QE (90 °C) pattern. This further highlights the difficulty of obtaining a pure sample of QE due to the very weak stability of the form when heated and explains why the mass loss in the desolvation step from the TGA data does not meet the theoretical loss of a stochiometric solvate.

### Crystal Structure Solution of the Quercetin Anhydrous Form (QA)

**Figure 7:**
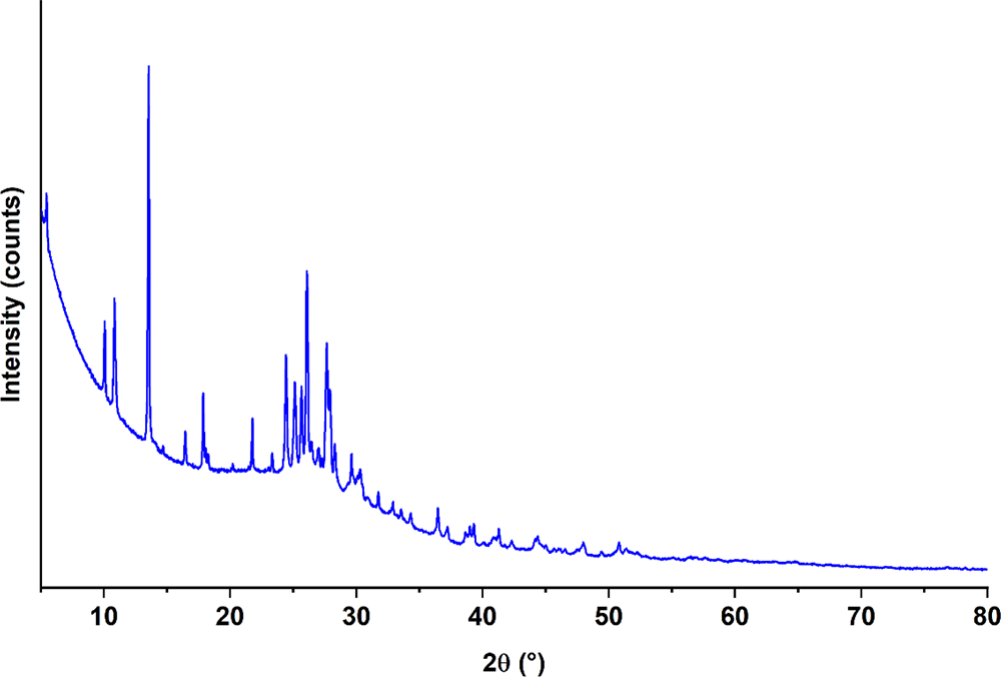
PXRD pattern of QA.

The anhydrous quercetin structure (QA) that resulted from the thermal desolvation of QE was solved from PXRD data collected in transmission mode, Figure 7, on the Rigaku Rint2500 diffractometer, using the EXPO software outlined in the methodology section. The crystallographic information and final Rietveld plot are reported in the Supporting Information, Table S1 and Figure S5, respectively.

**Figure 8:**
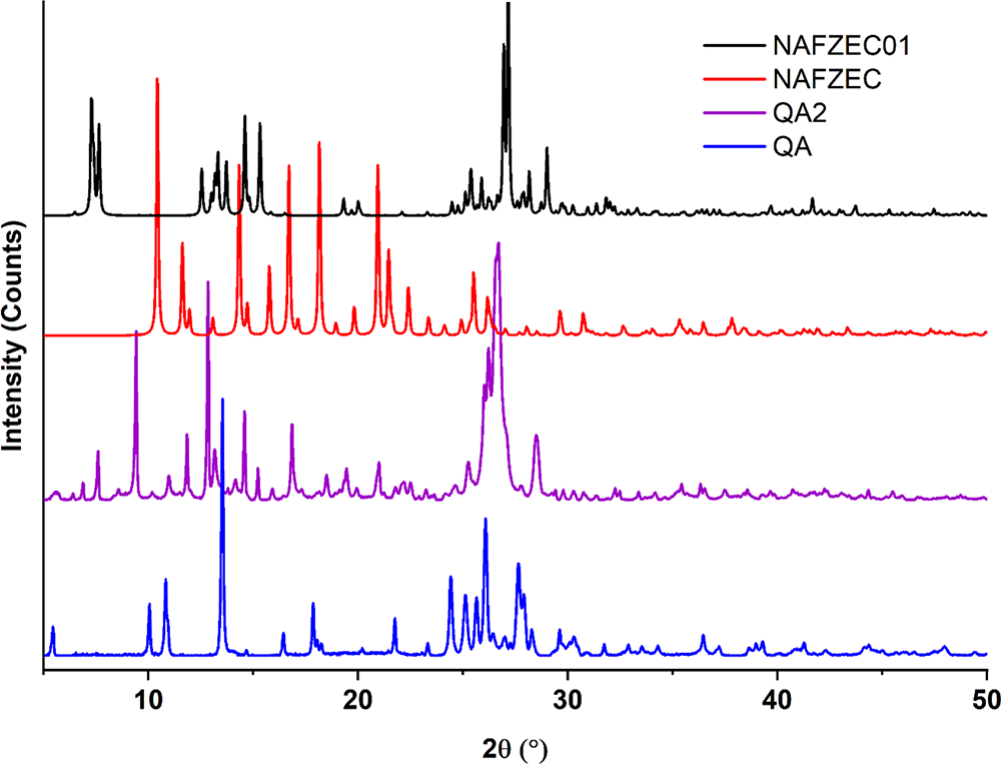
PXRD patterns of QA, QA2, and the two anhydrous quercetin polymorphs reported in the CSD.

It has been frequently reported in the literature that the desolvation of a solvated crystal form can provide an alternative pathway to the formation of polymorphic forms that would otherwise be difficult or impossible to crystallize by conventional crystallization techniques.^37^ It should be noted that the diffraction profile of QA does not match the dehydrated QDH or desolvated QDMSO (QA2) patterns previously obtained, nor to the PXRD patterns of the two anhydrous quercetin structures deposited in the literature.^6,14,24^ A PXRD pattern comparison between the different anhydrous quercetin patterns reported in the literature and the one obtained within this work is shown in Figure 8. However, the QA2 crystal structure remains unsolved.

**Figure 9:**
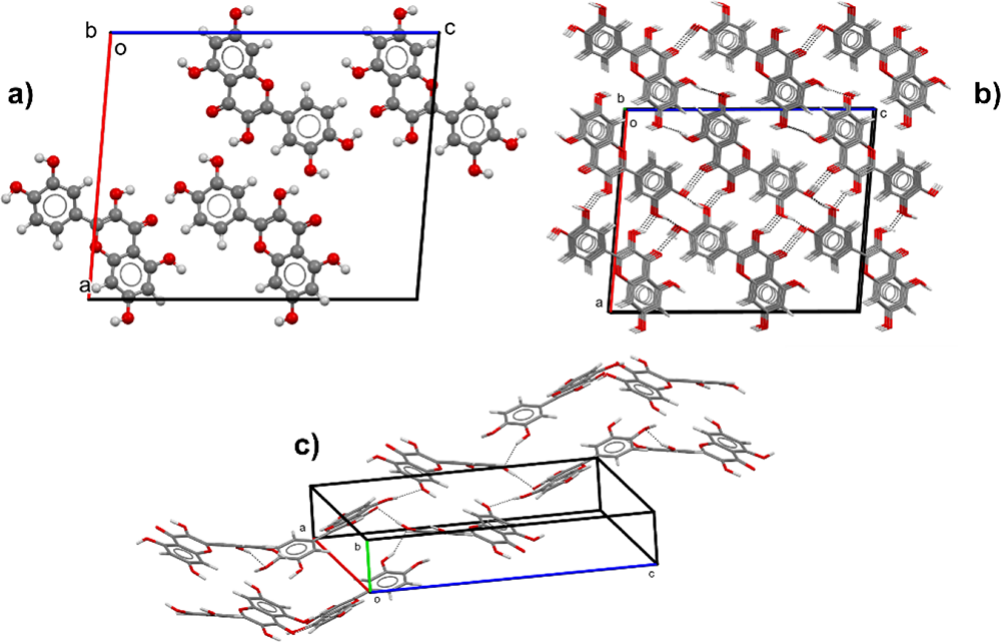
Packing diagrams of the QA form showing (a) the unit cell, (b) packing along the *b*-axis, and (c) hydrogen-bond pattern along the *a* + *c* direction. Black dotted lines indicate hydrogen-bond interactions.

The QA structure solved in this work is instead illustrated in the Supporting Information, Figure S6, and its structure packing is represented in Figure 9. The unit cell contains four quercetin molecules, each molecule forming one asymmetric unit (Figure 9a). Along the *b*-axis, the quercetin molecules are π-π stacked (Figure 9b) and the molecules are arranged in a zigzag motif along the *a* + *c* direction held by strong hydrogen-bond interactions between O-H donor groups and oxygen acceptor atoms of hydroxyl and carbonyl groups (Figure 9c). The dihedral angle between the phenyl and pyrone ring was found to be τ = 25.44(2)°, which makes the quercetin molecule more planar compared to the quercetin conformation of the previously reported anhydrous quercetin structure (τ_NAFZEC_ = 28.82°) and perhaps this facilitates the π-π stacking interactions along the *b*-direction.

### Solid-State NMR Spectroscopy Studies

**Figure 10:**
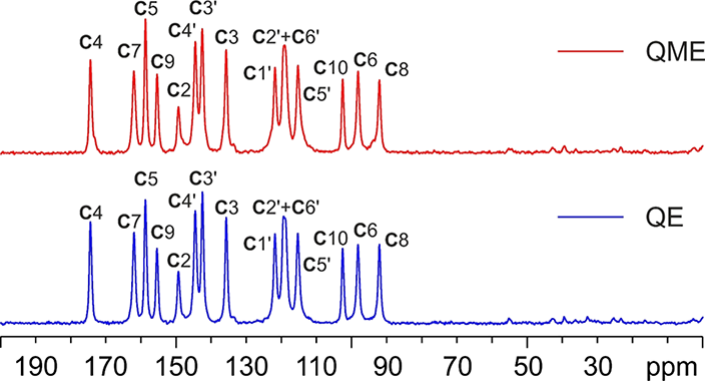
^13^C (100.61 MHz) CPMAS spectra of QE (in blue) and QME (in red), acquired at a spinning speed of 12 kHz at room temperature. Labels above peaks refer to assignments of the atoms of the quercetin molecule reported in Scheme 1.

The use of SSNMR plays an important role in unraveling structural features of solid crystalline materials, especially when such information cannot be obtained through diffraction analyses. Indeed, it allows assessing the purity of the samples and number of resonances in the ^13^C and ^15^N CPMAS SSNMR spectra and provides insights into the number of independent molecules in the unit cell (i.e., *Z*′). Additionally, the average full width at half maximum value of ^13^C signals is indicative of the degree of crystallinity of the material and the chemical shifts of the most significant resonances are able to suggest the protonation state of ionizable moieties and their involvement in hydrogen bonds.^43,44^ In this paper, we used ^13^C CPMAS SSNMR to characterize all the obtained quercetin samples, i.e., QE, QME, QA, and QA2. Figure 10 shows the ^13^C CPMAS spectra of QE and QME, with the assignment of the resonances, adopting the atom numbering presented in Scheme 1. An overlay of the QE and QME spectra is reported in the Supporting Information, Figure S7. All ^13^C chemical shifts are listed in Table S2 in the Supporting Information.

The two spectra are fully superposable, suggesting that the two samples contain the very same phase, or that they represent isomorphous phases. Moreover, they very well agree with those collected by Miclaus’ group for the two unstable solvates that they studied.^22^ The only difference in our spectra is the absence of any resonance ascribable to the presence of ethanol or methanol in QE or QME, respectively. In this sense, Miclaus’ spectra are characterized by two peculiarities: (a) in their QE spectrum, only the methyl signal appears but not the CH_2_ one; (b) the intensity of the methylic signal in QME does not fit that of the other signals suggesting the presence of a nonstoichiometric solvate or a very inefficient polarization transfer. We made several attempts, even with freshly prepared samples, to detect the ^13^C peak of methanol in QME also by means of a ^13^C direct excitation experiment (^13^C MAS) (not shown), which were unsuccessful. This led us to hypothesize that the two solvates are possibly nonstoichiometric and so unstable that they lose any trace of solvent during sample handling, leading to the obtainment of the same phase upon desolvation. This agrees with the nonstoichiometric relative intensity of the CH_3_ signal in the QME spectrum obtained by Miclaus and with the lower, than stoichiometrically expected, weight loss observed by TGA (see above). Nonetheless, the spectra clearly indicate that, in both cases, one independent molecule of quercetin in the unit cell is present.

**Figure 11:**
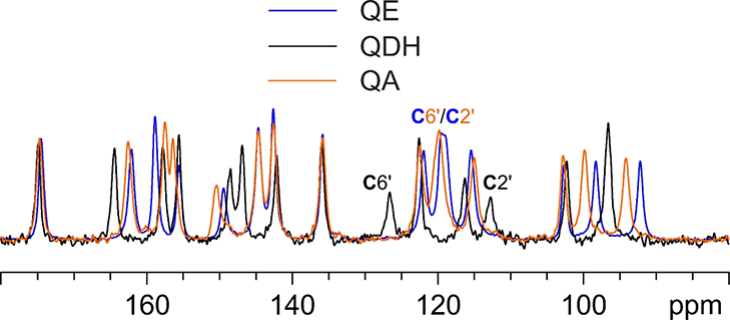
^13^C (100.61 MHz) CPMAS spectra of QE (in blue), QDH (in black), and QA (in orange), acquired at a spinning speed of 12 kHz at room temperature. Labels refer to the assigned C6′ and C2′ signals in all spectra (please, refer to Scheme 1 for atom numbering).

QE (taken as representative of both samples) was then compared to QA and to commercial QDH. The corresponding spectra are displayed in Figure 11 (the stacked spectra are reported in the Supporting Information, Figure S8).

The overlay of Figure 11 clarifies how the QE phase is different from both QDH and QA. Additionally, the spectra of QDH and QA well agree with those previously reported for the same commercial crystal forms. Earlier studies performed by Olejniczak and Potrzebowski^20^ and Filip et al.^21^ suggest that, while in QDH quercetin displays an *anti* conformation, in QE, QME, and QA, it adopts the *syn* one. This information can be mainly assessed by the chemical shifts of C2′ and C6′, which, in the case of the *syn* conformer, tend to converge to about 120 ppm, while, in the *anti* one, are well separated, falling at about 127 (C6′) and 113 (C2′) ppm.

Regarding QA, we compared our results with those obtained by Vasisht’s group;^6^ despite many crystallization attempts, we were never able to obtain the same PXRD pattern as theirs, while consistently achieving the one shown in this paper, which perfectly reproduces that of QA, previously studied by Filip’s group.^21^ Moreover, the ^13^C CPMAS spectrum that Vasisht proposes does not coincide with that of QA. This leads to hypothesize that the crystal structure deposited in the CSD (refcode: NAFZEC) by Vasisht either represents a “disappearing polymorph” of anhydrous quercetin, or that it was solved starting from a physical mixture of several phases, as the corresponding ^13^C CPMAS SSNMR spectrum seems to suggest. On the contrary, the structure presented in this work is representative of the anhydrous quercetin reported by Filip, which was never deposited in the CSD. This is further endorsed by the DFT optimization of the QA and NAFZEC crystal structures, which confirmed the higher stability of our structure with respect to Vasisht’s. As known, the maximum energy difference between polymorphs is usually of 10 kJ/mol, while unexpectedly, the energy of NAFZEC resulted to be +46.65 kJ/mol higher than QA. Then, we proceeded to the comparison of the experimental and optimized structures of QA and NAFZEC (Table 2).

**Table 2:** Comparison of the Cell Parameters of the Experimental (EXP) and Optimized (OPT) Crystal Structures of QA and NAFZEC^a^

parameter	QA EXP	QA OPT	NAFZEC EXP	NAFZEC OPT
space group	*P*2_1_/*c*	*P*2_1_/*c*	*Pn*21*a* (33)	*Pn*21*a* (33)
*Z*, *Z*′	4, 1	4, 1	4, 1	4, 1
*a*/Å	16.2752	16.2633	14.7998	15.4280
*b*/Å	3.7155	3.5841	11.2379	8.0385
*c*/Å	19.9723	19.6573	10.3512	10.9332
α°	90.0000	90.0000	90.0000	90.0000
β°	94.7275	93.0266	90.0000	90.0000
γ°	90.0000	90.0000	90.0000	90.0000
*V*/Å^3^	1203.63	1144.10	1721.60	1355.91
%Error on volume	–5.2	–26.96		
RMSD_20_	0.213	1.161		

a %Error on cell volume and RMSD20 are also reported.

As can be seen from Table 2, for the QA structure the volume, the experimental and computed unit cell parameters are in perfect agreement. The %Error on the cell volume of −5.2% is consistent with the average %Error typically found between experimental and computed cell volumes, i.e., the computed structure is subjected to cell-shrinkage as the optimization is performed at 0 K. The overlay of the experimental and optimized crystal structure of QA is reported in the Supporting Information, Figure S9. On the other hand, the optimization of the NAFZEC crystal structure shows a dramatic reduction of the cell volume (−26.96%) with a retention of the space group. To check the goodness of the optimization result, the experimental structure was optimized using also the VASP package with the PBE-pseudopotentials. Two attempts were made, using the TS and MBD methods for dispersion correction. Both optimizations converged to the same result of the first one. The predicted molecular volume for quercetin, using the formula by Hofmann^45^ is about 339 Å^3^. Thus, the estimated cell volume for quercetin, in a *Pn*21*a* space group (*Z*, *Z*′ = 4, 1), should be of 1354 Å^3^. Indeed, the experimental structure shows some voids in the unit cell (Figure S10), which are not consistent with the close packing usually found in organic molecular crystal structures.

**Figure 12:**
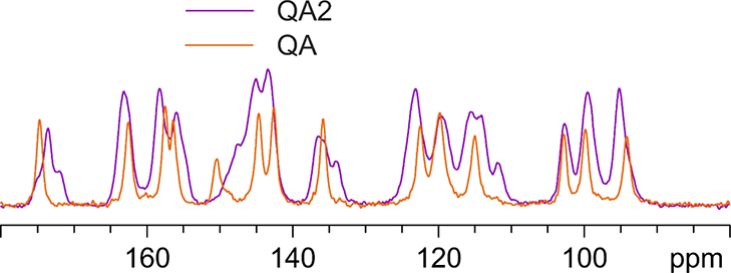
^13^C (100.61 MHz) CPMAS spectra of QA (in orange) and QA2 (in purple), acquired at a spinning speed of 12 kHz at room temperature.

Finally, a ^13^C CPMAS measurement was performed on the QA2 solid form, obtained by desolvating QDMSO crystals,^13^ too. Figure 12 shows a comparison between its ^13^C CPMAS SSNMR spectrum and that of the solved quercetin anhydrous phase (QA). The peak assignments are included in the Supporting Information, Table S2.

The QA2 phase appears different from the QA phase. Unfortunately, due to strong peak overlapping, no definitive insight into the number of independent molecules in the unit cell can be reached, but by a process of peak fitting and integration of the carboxylic region (180–170 ppm), we hypothesize that the new phase contains four quercetin molecules in the asymmetric unit.

### Stability Studies

**Figure 13:**
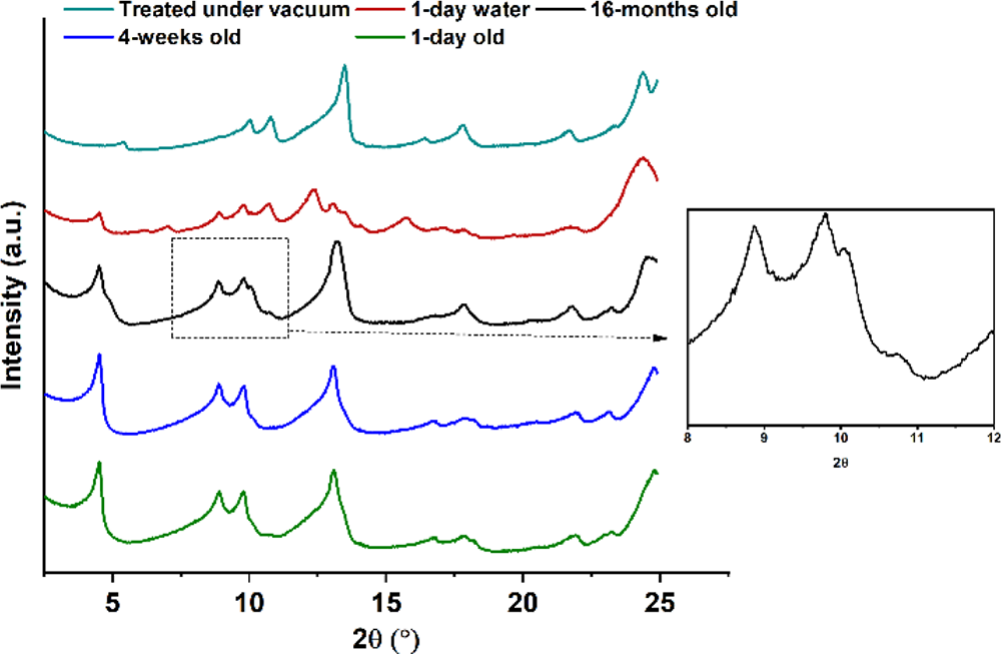
SAXS/WAXS patterns of QE samples treated under different conditions.

Samples of QE of different age and processing history were compared to study the stability of the crystal structure in atmospheric conditions. The SAXS/WAXS data of the analyzed samples are shown in Figure 13.

The QE samples that were 1-day, 4-weeks, and 16-months old were left in open vials in the laboratory at room temperature (20 °C) and pressure. The results show that the patterns for the 1-day and 4-weeks old samples were identical; therefore, QE is unlikely to transform over such period of time. However, the 16-months old sample exhibited some extra peaks at 10.1° and 10.7° (2θ), and the peak around 13.0° appears to be slightly shifted to the right compared to the other patterns. These extra peaks match with those of the desolvated form of QE shown earlier. As the pattern appears to confirm a mixture of QE and of its desolvated form, it shows that, over the period of 16 months, QE is likely to slowly desolvate when the samples are stored at room temperature (20 °C).

The pattern of a QE sample that was slurried in pure water for 24 h contained peaks that are characteristic of QE, but also some extra peaks at 10.8°, 12.9°, 13.9°, and 14.2° (2θ), which are characteristic peaks of QDH. This suggests that, when the QE form is slurried in water, it can transform back into the QDH form, which aligns with the observation that, in such a high water activity, the QDH is the thermodynamically stable form. However, the pattern suggests that the transformation is incomplete in 24 h and that the sample is a mixture of both QE and QDH, as it contains characteristic peaks of both forms. This shows that, for applications of quercetin in water, QE would not be a stable solid form as it would transform to QDH.

The pattern of the QE sample treated in vacuum for 24 h exhibits peaks at 5.5°, 10.2°, 10.9°, and 13.5° (2θ) that completely match the peaks of the desolvated QE obtained by heating the sample to 90 °C. The pattern does not contain any peaks from the original QE form; therefore, the desolvation in vacuum appears to be complete and gives a pure desolvated QE form (QA). Therefore, it can be concluded that the desolvation of QE can be accelerated either by heating the sample at a temperature above 28 °C, as this was the onset of desolvation from the TGA/DSC experiments, or by treating the solid in vacuum for 24 h.

### Solubility Data of Quercetin Solid Forms

**Figure 14:**
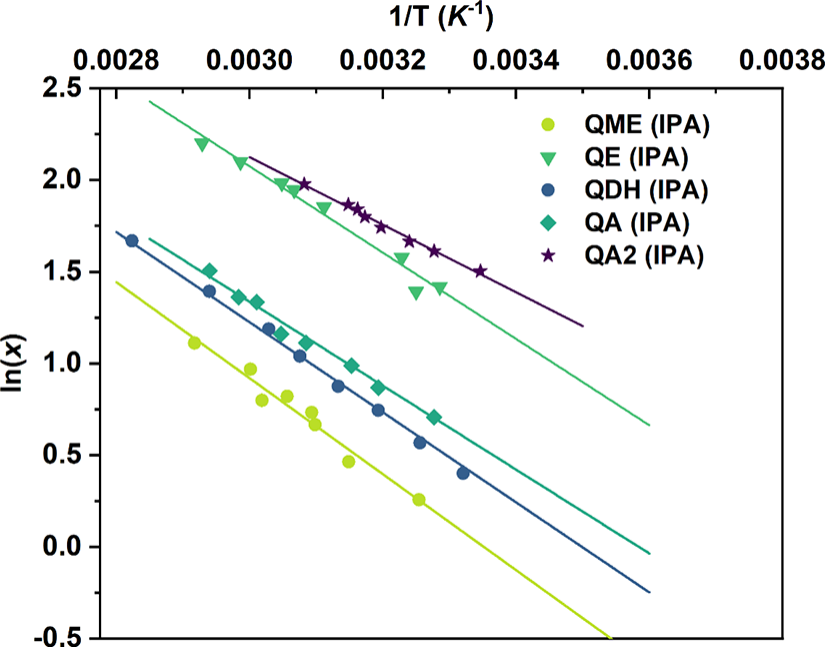
Solubility data of quercetin solid forms collected in isopropanol (IPA). The van’t Hoff plots are represented by the lines.

Figure 14 shows a plot of the solubility of the different quercetin solid forms in isopropanol. The solubility of pure compounds was determined for temperatures between 20 and 70 °C. The results could be well correlated with the van’t Hoff equation (eq 1) as shown from the linear fit in Figure 14
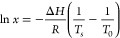
1

From the obtained solubility data, we can observe that all the quercetin solid forms studied in this work have different solubility values, indicating that they all represent different crystal structures. Interestingly QE and QME have different solubilities despite presenting similar PXRD patterns. The two anhydrous forms, QA and QA2, show very different solubility values, confirming that these are two different polymorphs of pure quercetin. As QA has a lower solubility than QA2, it can be assumed that this form is the more stable anhydrous polymorph. It is worth noticing that, in isopropanol, the less soluble form is the QME, while QA2 is the most soluble one.

**Scheme 2:**
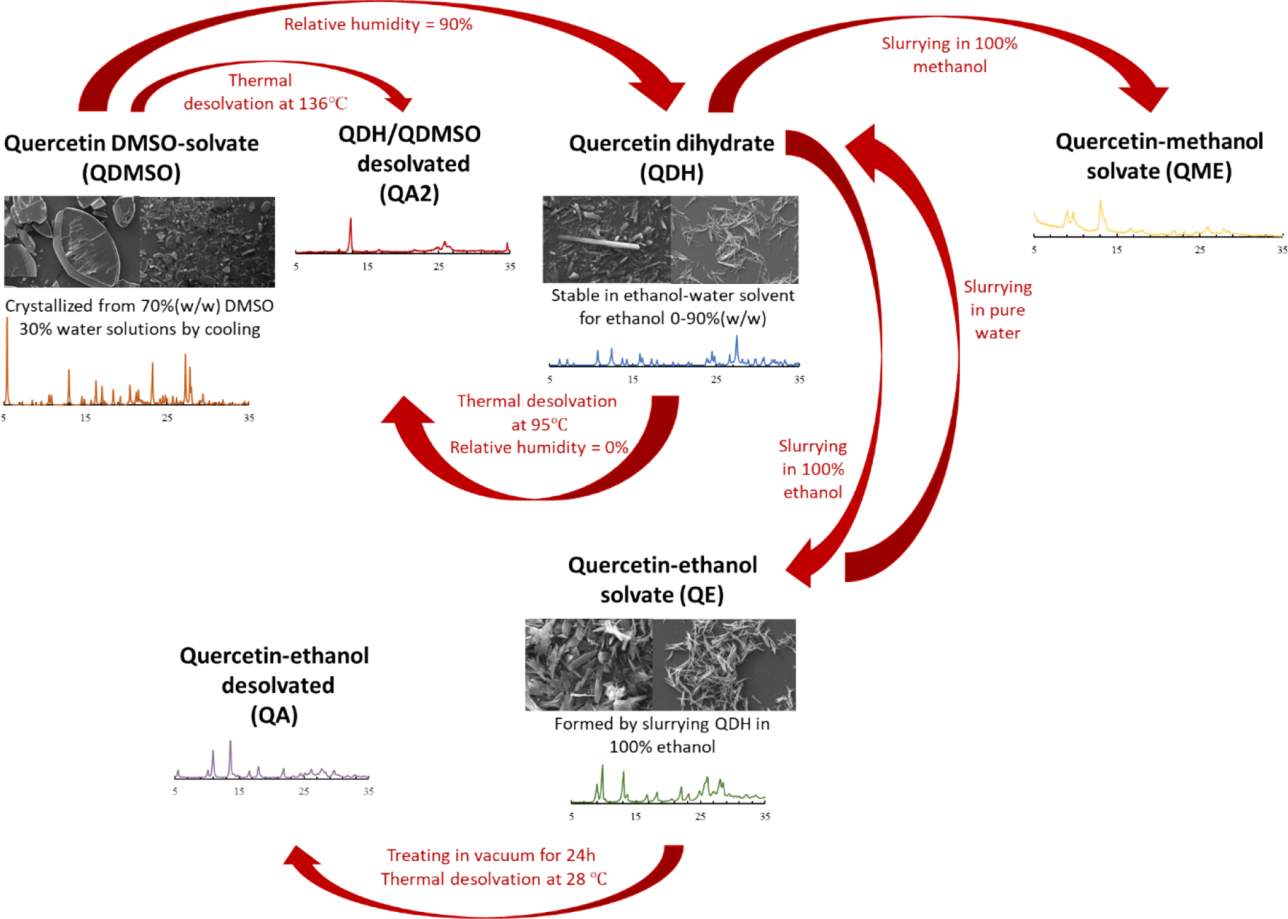
Solid-Form Landscape of Quercetin, Including the Dihydrate (QDH), DMSO-Solvate (QDMSO), Ethanol-Solvate (QE), and Methanol-Solvate (QME) Forms and Their Desolvated Forms (QA and QA2)

When using solid forms of quercetin for various applications in the nutraceutical or food industry, it is of critical importance to have a knowledge of the solid-form landscape of the substance. Scheme 2 summarizes the solid-form landscape of quercetin, showing the different structures of quercetin and transformations between them, based on the work done in this paper and in our previous publications.^14^^,^^26^

## Conclusions

Recrystallization of QDH by slurrying in pure ethanol and pure methanol resulted in the formation of the unstable intermediates QE and QME, which are probably crystal structures including ethanol and methanol, respectively, bound to the quercetin molecules in their structures. The two forms are characterized by weak solvate stability and slow desolvation at room temperature (20 °C). The thermal analyses showed that the QE form loses all the ethanol at an onset temperature of 28.5 °C to form its desolvated structure, which is a stable structure of anhydrous quercetin (QA).

The QA crystal structure was solved from PXRD data using the EXPO software in the *P*2_1_/*c* space group and was found to contain four quercetin molecules in the unit cell. This quercetin anhydrous form (QA) is different from the desolvated quercetin structure (QA2) that can be obtained by desolvation of QDMSO solvate, and from any anhydrous quercetin structure previously reported. However, the crystal structure of QA2 is still unknown. The SSNMR and computational studies confirm the goodness of the structure of the anhydrous polymorph. Stability studies on QE revealed that other pathways to the formation of the QA include treating the QE form in vacuum for 24 h, or slow solid-state transformation over a period of more than 16 months at ambient temperature and pressure.

These experimental findings enhance the knowledge around the different solid forms of this important bioflavonoid substance. A comprehensive understanding of the physicochemical properties, crystallization conditions, and transformation between the various forms is essential when designing processes and optimal solid forms for specific applications using quercetin.

## Supporting Information Available

SAXS/WAXS patterns of QDH ethanol slurry experiments; SEM image of QDH crystals from the 70/30%(w/w) ethanol/water solvent ratio; QE mass-loss over time experiments; crystallographic data collection and structure refinement parameters of the QA crystal structure; Rietveld plot of the QA cystal form; QA CIF and checkCIF files; SSNMR data of QME, QE, QDH, QA, and QA2 crystal forms; DFT crystal structure optimization of the QA crystal structure; and void calculation figure of the NAFZEC crystal structure (PDF)
