# Supplementary material for: Navigating the Complex Solid Form Landscape of the Quercetin Flavonoid Molecule
Source: Cryst Growth Des. 2023 Jul 13;23(8):6034–45. doi: 10.1021/acs.cgd.3c00584 (PMC10401642; doi:10.1021/acs.cgd.3c00584)
Supplement: Supplementary file 1 — cg3c00584_si_001.pdf [file cg3c00584_si_001.pdf]

SUPPORTING INFORMATION FOR:

NAVIGATING THE COMPLEX SOLID FORM LANDSCAPE OF THE QUERCETIN FLAVOINOID MOLECULE

Panayiotis Klitou[1], Emmanuele Parisi[2], Simone Bordignon[3], Federica Bravetti[3], Ian Rosbottom[4], Marzia Dell'Aera[5], Corrado Cuocci[5], Michele R. Chierotti[3], Angela Altomare[5], Elena Simone[2,1]*

[1]School of Food Science and Nutrition, Food Colloids and Bioprocessing Group, University of Leeds, Leeds, LS2 9JT UK

[2] Department of Applied Science and Technology (DISAT), Politecnico di Torino, Torino, 10129, Italy

[3] Università degli Studi di Torino, Dipartimento di Chimica I.F.M, Via P. Giuria 7, Torino, 10125, Italy

[4]Materials Science, Chemical Development, GSK, Gunnels Wood Road, Stevenage, SG1 2NY, UK

[5] Institute of Crystallography IC – CNR, via Amendola 122/O, 70126 Bari, ITALY

*Corresponding author: elena.simone@polito.it

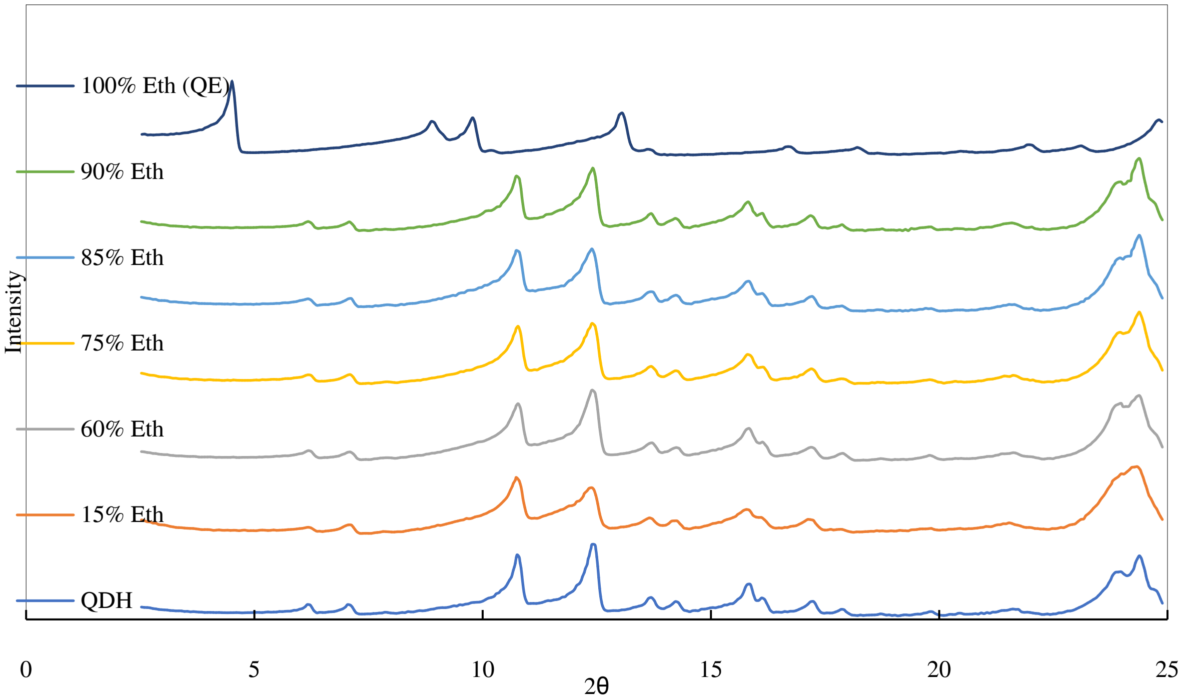

Figure S1. SAXS/WAXS patterns for samples from slurrying experiments for solvent ratios from 15%(w/w) ethanol to 100% ethanol.

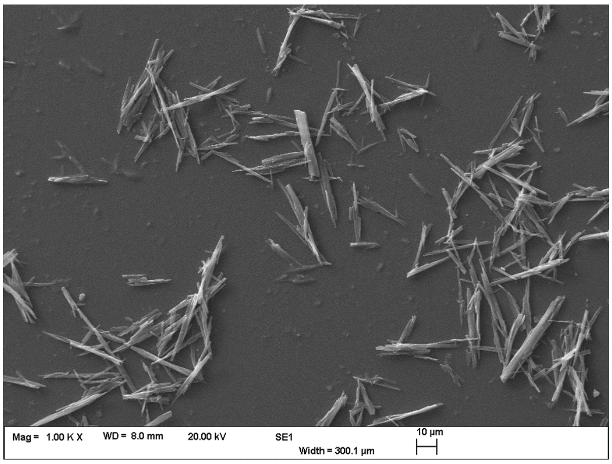

Figure S2. SEM image of QDH crystals from 70% ethanol (w/w) 30% water solvent.

QE mass loss over time experiments

Dynamic Vapor Sorption Experiment. The results from the DVS experiment are shown in Figure S3. The data shows that there is a decrease in the mass of the sample for approximately the first 30 minutes, which should be due to the evaporation of the liquid ethanol from the slurry. After that, the mass of the sample appears to be constant. At time t=100 min, the recorded rate of mass change is -0.004 %/min, and after t=288 min the rate becomes 0 %/min. This shows that a time of approximately 300 minutes was enough to dry the specific sample completely and after that no further loss of mass was recorded.

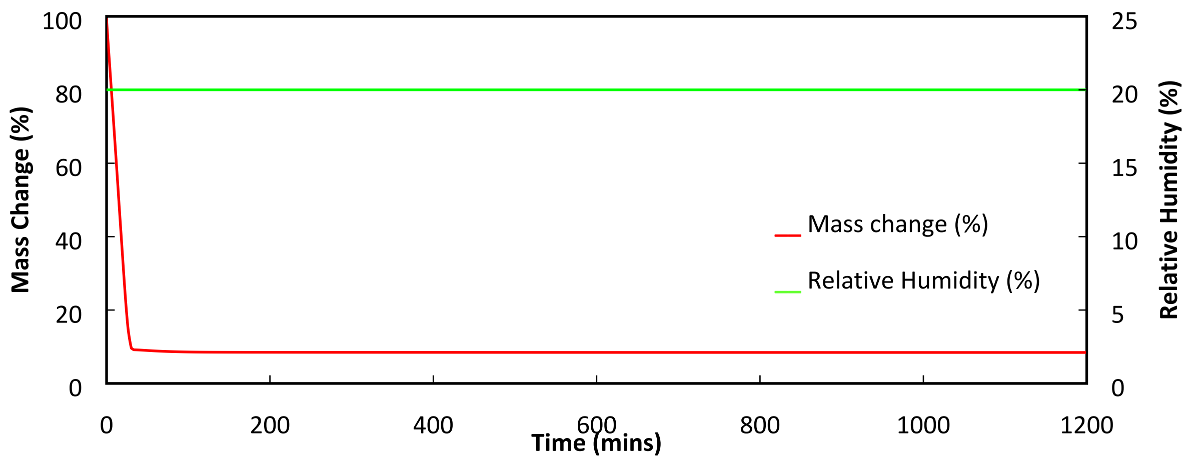

Figure S3. DVS data for QE slurry at constant temperature of 20°C and relative humidity of 20%.

Monitoring mass sample of QE over time: The results of this experiment, illustrated in Figure S4, show that the mass of the sample decreases considerably from day 0, which is the day the sample is filtered, to day 1. This initial loss is due to the sample still being wet with ethanol after filtration and the mass loss is due to the evaporation of ethanol. It is assumed that the loss is only due to drying, as suggested by the X-ray data which show that the structure does not change within a time of 4 weeks (See Stability Studies Section). However, after day 1 the mass appears to be fairly stable. Assuming that the sample is dry on day 1, and that being 100% of the mass, the mass change from day 1 to day 6 is found to be 0.8%. The change in mass is not considerable to be associated with any de-solvation event of the sample during the specific timeframe.

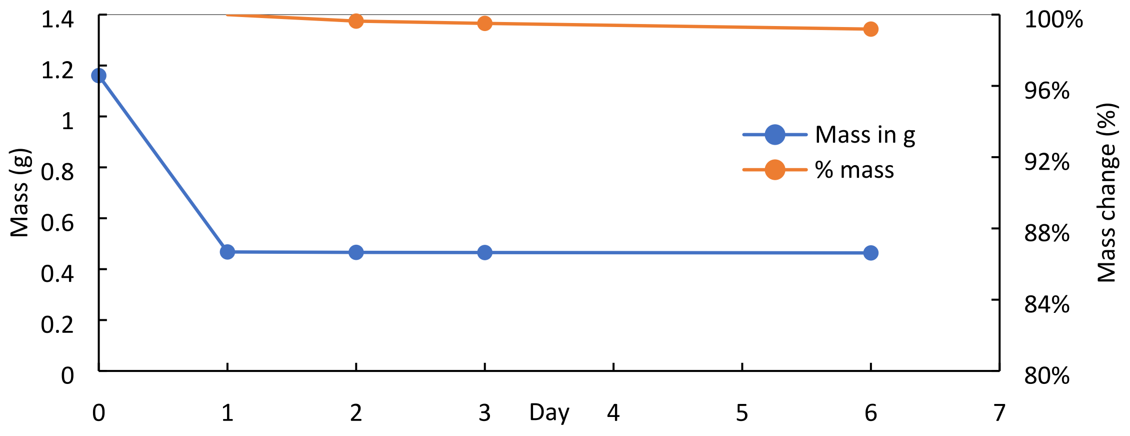

Figure S4. QE mass loss over time in laboratory conditions

X-ray Crystallographic Data Collection and Structure Refinement for 2-(3,4-dihydroxyphenyl)-3,5,7-trihydroxy-4H-chromen-4-one (quercetin)[1]

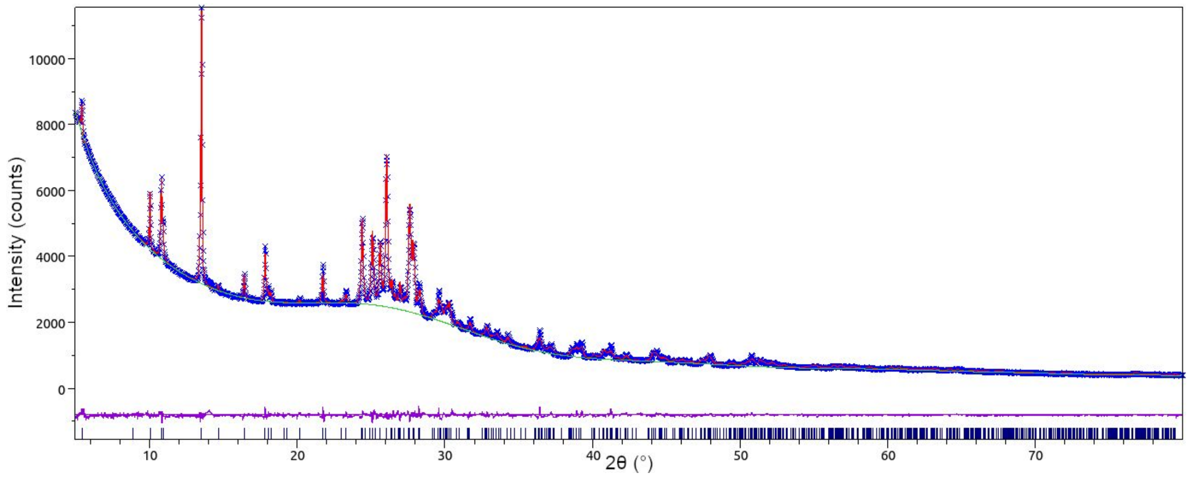

Figure S5. Final Rietveld plot. Observed data points are indicated by crosses, the best-fit profile (upper trace), the difference pattern (lower trace) and the background are solid lines. Vertical bars indicate the positions of Bragg peaks.

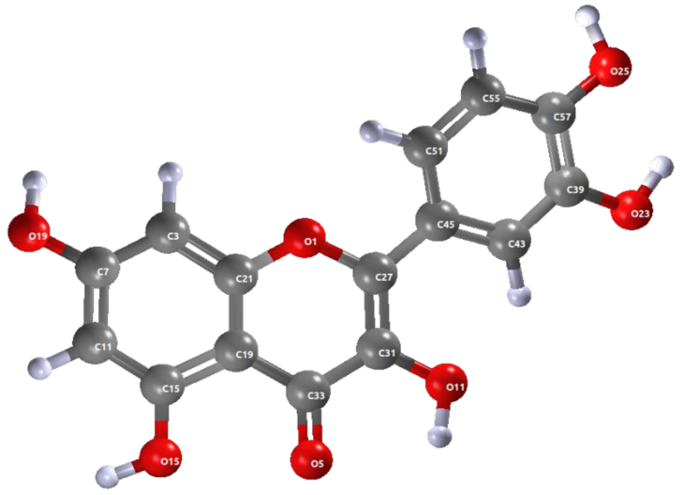

Figure S6. The asymmetric unit of the title compound with the atom labelling scheme for non-H atoms. Color legend: carbon (light grey), hydrogen (white), oxygen (red).

Table S1. X-ray Crystallographic Data Collection and Structure Refinement parameters of the title compound.

**Crystal data**

Chemical formula	$C_{15}H_{10}O_7$
Formula weight	302.24
Crystal system	Monoclinic
Space group	$P\,2_1/c$ (No. 14)
Temperature (K)	293
a, b, c (Å)	16.275 (2), 3.7155 (4), 19.972 (3)
β (°)	94.727 (5)
Volume (Å^3)	1203.6 (3)
Z	4
Z'	1
Radiation type	Cu *Kα* radiation, λ = 1.540560 Å
**Data collection**	
Diffractometer	Rigaku RINT2500
Specimen mounting	special glass capillary
Data collection mode	transmission
2θ (°)	$2\theta_{min}$ = 5.00, $2\theta_{max}$ = 80.00
**Structure solution**	
Methods	Direct space method, direct methods
Parameters	6+3
Cost function	4.87
**Refinement**	
R_p	1.493
R_{wp}	2.334
R_{exp}	2.360
R_{Bragg}	7.035
χ^2	0.978
No. of data points	3751
Profile function	Pearson VII
Refinement parameters	
Lattice	4
Positional	66
Thermal	2
Profile	10
Background	17

Peak-shift	3
Restraints	24
H-atom treatment	H-atom parameters constrained
**Programs**	
Indexing	N-TREOR09, DICVOL04
Space group determination	EXPO2014
Structure solution and refinement	EXPO2014
Model building	NAFZEC[2] from CSD, MOPAC2016
Structure validation	Quantum ESPRESSO

SSNMR data

Table S2 ^{13}C ss-NMR chemical shifts of the signals in the ^{13}C CPMAS spectra of QME, QE, QDH, QA and QA2, with relative assignments (please, refer to Scheme 1 of the manuscript for C atom numbering).

QME	
**^{13}C SSNMR chemical shift (ppm)**	**Assignment**
174.5	C4
162.2	C7
158.9	C5
155.5	C9
149.4	C2
144.7	C4'
142.6	C3'
135.9	C3
122.0	C1'
119.5	C2'+C6'
115.4	C5'
102.6	C10
98.3	C6

92.2	C8
**QE**	
**^{13}C SSNMR chemical shift (ppm)**	**Assignment**
174.5	C4
162.2	C7
158.9	C5
155.5	C9
149.4	C2
144.6	C4'
142.6	C3'
135.8	C3
121.9	C1'
119.4	C2'+C6'
115.4	C5'
102.6	C10
98.3	C6
92.2	C8
**QDH**	
**^{13}C SSNMR chemical shift (ppm)**	**Assignment**
174.8	C4
164.4	C7
157.7	C9
155.5	C5
148.4	C2
146.8	C4'
142.1	C3'
135.9	C3
126.6	C6'
122.5	C1'
116.2	C5'
112.7	C2'
102.2	C10
96.5	C6+C8
**QA**	
**^{13}C SSNMR chemical shift (ppm)**	**Assignment**
174.7	C4
162.5	C7
157.4	C9
156.4	C5
150.3	C2
144.6	C4'
142.5	C3'
135.8	C3

122.4	C1'
119.8	C2'+C6'
114.9	C5'
102.7	C10
99.8	C6
94.1	C8
**QA2**	
**^{13}C SSNMR chemical shift (ppm)**	**Assignment**
175.0	C4
173.5	C4
172.0	C4
163.1	C7
158.2	C9
155.9	C5
147.4	C2
145.0	C4'
143.4	C3'
136.4	C3
135.8	C3
134.1	C3
123.1	C1'+C6'
119.5	C2'+C6'
115.5	C5'
114.0	C5'
111.9	C2'
102.6	C10
99.5	C6
95.1	C8

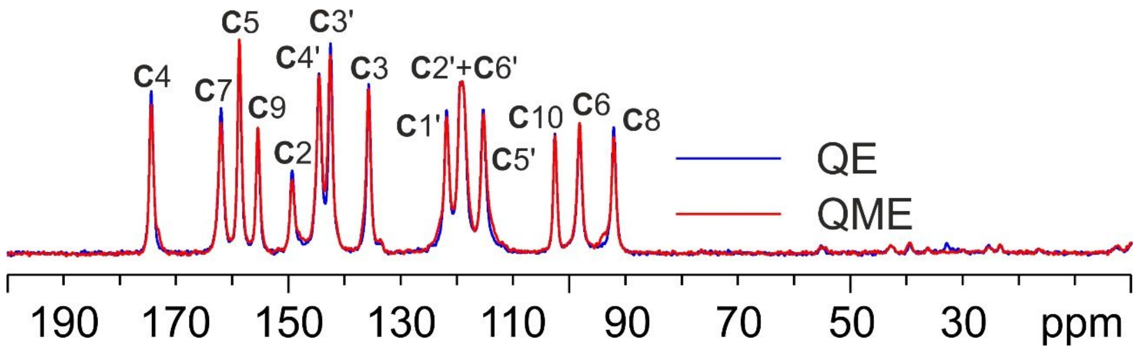

Figure S7 ^{13}C (100.61 MHz) CPMAS spectra of QE (in blue) and QME (in red), acquired at a spinning speed of 12 kHz at room temperature. Labels above peaks refer to assignments of the atoms of the quercetin chart reported in Scheme 1 of the manuscript.

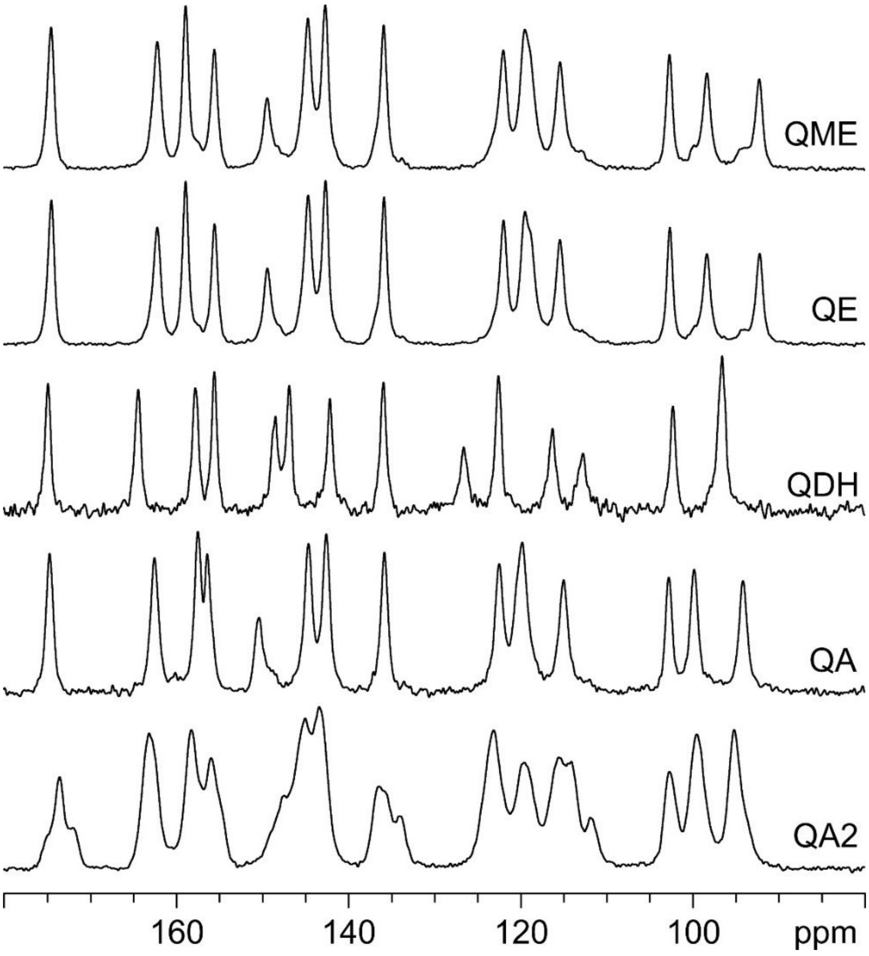

Figure S8 ^{13}C (100.61 MHz) CPMAS spectra of QME, QE, QDH, QA and QA2 with a spinning speed of 12 kHz at room temperature.

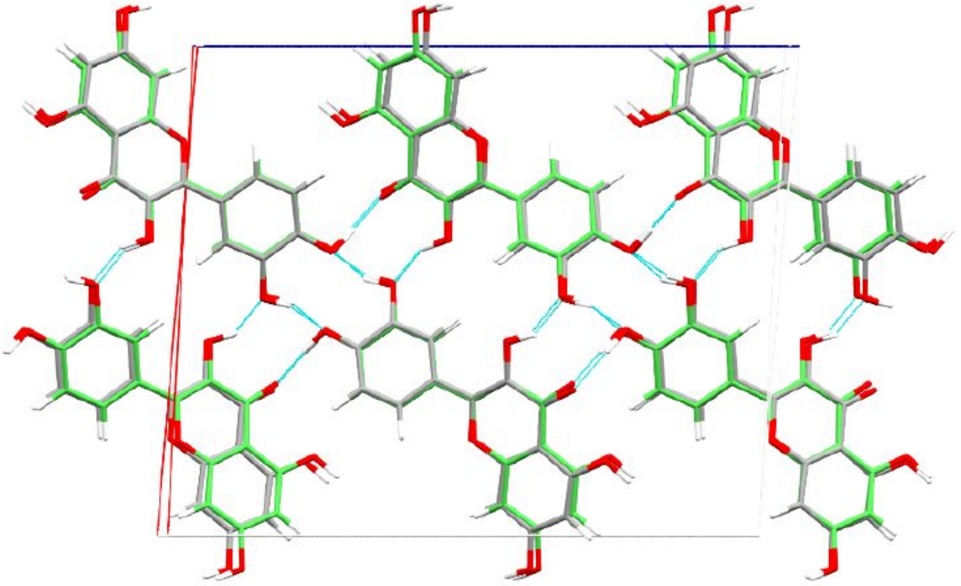

Figure S9 Overlay of the experimental and optimised crystal structures of QA. View direction [010].

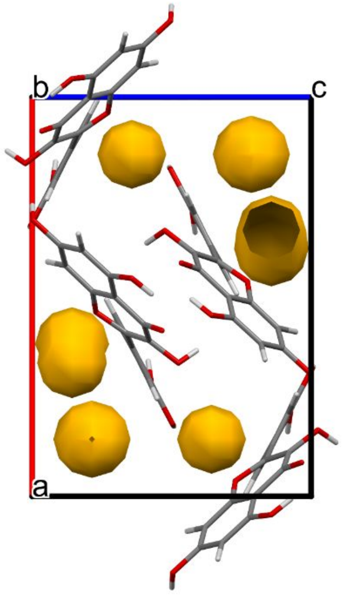

Figure S10 Experimental unit cell of NAFZEC.[2] View direction [010].

REFERENCES

[1] X-ray Crystallographic Information File QA.cif contains the supplementary crystallographic data for this paper, and is supplied as independent Supporting Information file for this article. This file can also be obtained free of charge from the Cambridge Crystallographic Data Centre via www.ccdc.cam.ac.uk/data_request/cif (CCDC 2248118).

[2] K. Vasisht, K. Chadha, M. Karan, Y. Bhalla, A. K. Jena, and R. Chadha, "Enhancing biopharmaceutical parameters of bioflavonoid quercetin by cocrystallization" (2016). CrystEngComm, 18, 8, 1403–1415, doi: 10.1039/C5CE01899D.
